# Waist-to-Hip Ratio, Cardiovascular Outcomes, and Death in Peritoneal Dialysis Patients

**DOI:** 10.4061/2010/831243

**Published:** 2010-07-05

**Authors:** Winnie S. Su, Catherine M. Clase, K. Scott Brimble, Peter J. Margetts, Trevor J. Wilkieson, Azim S. Gangji

**Affiliations:** ^1^Division of Nephrology, McMaster University, St. Joseph's Healthcare, Hamilton, ON, Canada L8N 4A6; ^2^Department of Clinical Epidemiology and Biostatistics, McMaster University, Hamilton, ON, Canada L8N 4A6

## Abstract

*Objectives*. The primary objective of this study was to determine the relationship between waist-to-hip ratio (WHR), cardiovascular (CV) events, and mortality in peritoneal dialysis (PD) patients. A secondary objective was to investigate the association between abdominal obesity and systemic inflammatory markers. *Methods*. This is a prospective study of 22 prevalent PD patients. WHR was measured at baseline. C-reactive protein (CRP), tumour necrosis factor-*α* (TNF-*α*), and interleukin-6 (IL-6) were measured. Main outcomes were first CV event and death from all causes. Survival analysis was used to examine the relationship between anthropomorphic measures and clinical outcomes. *Results*. Mean follow-up period was 3.1 years. In Kaplan-Meier analysis, survival was lower in those with higher WHR (*P* = .002). In Cox regression, WHR independently predicted mortality and first CV event after adjustment for known ischemic heart disease (hazard ratio [HR] 1.17, confidence interval [CI] 1.05–1.30 for death; HR 1.13, CI 1.01–1.26 for CV event). WHR correlated with serum TNF-*α* (*r* = 0.45; *P* = .05). *Conclusion*. The results of this study suggest WHR may be a risk factor for increased CV events and mortality in PD patients. Abdominal obesity is also associated with inflammatory markers. Larger studies are warranted to confirm these findings.

## 1. Introduction

Cardiovascular (CV) disease is the leading cause of death in dialysis patients [1]. Obesity increases the risk of CV disease in the general population [2]. In particular, abdominal obesity as measured by waist-to-hip ratio (WHR) has emerged as a better predictor of CV risk than body mass index (BMI) [3]. In several large epidemiologic studies, WHR was an independent predictor of coronary artery disease and death [3–5]. 

Studies on abdominal obesity in patients with chronic kidney disease are limited. In one study of patients with mild to moderate chronic kidney disease (CKD), increased WHR predicted fatal and nonfatal coronary artery disease events [6]. The first study of WHR in hemodialysis (HD) patients was recently published in [7]. Higher WHR and waist circumference (WC) predicted CV and all-cause death after adjusting for BMI whereas higher BMI was protective [7]. To our knowledge, there are no studies in peritoneal dialysis (PD) patients that have assessed the relationship between WHR and clinically important adverse events. 

One of the postulated mechanisms for abdominal obesity's detrimental effect is its relationship with inflammatory markers, and inflammation is associated with increased CV risk in chronic kidney disease [8, 9]. Excess abdominal fat has been shown to be associated with increased C-reactive protein (CRP), tumor necrosis factor-alpha *(*TNF-*α*), and interleukin-6 (IL-6) in general populations [10, 11], but studies in PD patients are scarce [12]. 

The primary objective of this study was to investigate the relationship between central obesity as measured by WHR, and CV events and death in PD patients. A secondary objective was to determine if there was an association between anthropomorphic measures and systemic inflammatory markers.

## 2. Methods

This is a prospective study of 22 prevalent, stable, and consenting PD patients who were recruited from one of the affiliate teaching hospitals associated with McMaster University, (St. Joseph's Healthcare), Hamilton, Canada. Study protocol was approved by the St. Joseph's Healthcare Research Ethics Board. Patients were included in the study if they were 18 years of age or older and on PD for at least 3 months. Exclusion criteria included a history of peritonitis in the previous month and inability to provide informed consent. 

Patient charts were reviewed to collect demographic information, a list of comorbidities, medications, and dialysis prescription. Transport property of the peritoneal membrane was determined by the fast peritoneal equilibration test [13], and transport status was established based on the four-hour dialysate/plasma (d/p) creatinine. Residual renal function was calculated as the mean of 24-hour urea and creatinine clearances.

On the day of testing, two hours into the dwell for the peritoneal equilibration test, serum IL-6, TNF-*α*, and CRP (Cardiophase* hs CRP Dade Behring Inc. Newark, DE) levels were drawn. IL-6 and TNF-*α* were measured by ELISA (R&D Systems, Minneapolis MN). 

 Weight and height were measured without shoes. Measurements and patient assessment were completed after patients had drained their overnight dialysate dwell. Waist circumference (WC) was measured around the narrowest point between the coastal margin and the iliac crest. Hip circumference was measured at the level of the widest diameter around the gluteal region. Waist-to-hip ratio is waist circumference divided by hip circumference. BMI is weight divided by (height)^2^. 

Patients were recruited between March and September of 2005. They were followed until December 2008 or death. The main outcomes were first CV event (myocardial infarction [MI], stroke, amputation, cardiac revascularization, or CV death); only one event was included for the analysis and death from all causes. MI was diagnosed when two out of three of the following criteria were met: typical symptoms, elevation in cardiac enzyme (troponin T > 0.1 mcg/L), or diagnostic changes on the electrocardiogram. Stroke was defined as new neurologic deficit thought to be vascular in origin and lasting more than 24 hours. CV death included death caused by MI, stroke, complications of peripheral arterial disease, and all sudden death. 

## 3. Statistics

All analyses were performed with the statistical package, SPSS (version 17; SPSS Inc. Chicago, IL). Subjects were divided in to two groups according to the median value for their gender for each anthropomorphic measure. Mann-Whitney *U* test was used to compare continuous baseline variables between groups. Fisher exact test was used to compare categorical CV risk factors between groups. Bivariate correlations are reported using the Spearman rank correlation coefficient. Two-sided probability (*P*) values were calculated with statistical significance set at alpha ≤.05. Univariate comparisons of survival between those with high or low WHR were performed with Kaplan-Meier analysis and log-rank tests. The relationships between WHR and clinical outcomes were also investigated using Cox regression. Logistic regression was used if assumption of proportionality was violated. Baseline variables found to be significantly different between high and low WHR groups were entered into a Cox regression model as covariate. However, the number of variables entering the model was limited by the number of events to ensure model stability [14]. 

## 4. Results

All 43 eligible patients at our centre were approached, and 22 patients consented. The majority of the patients declined due to time commitment and need for followup. All enrolled patients were followed to the end of the study.

Baseline characteristics of the 22 patients are summarized in Table 1. Mean age ± standard deviation was 61 ± 16 years; 59% were men, and 95% were Caucasian. At baseline, 37% had diabetes (DM), and 23% had known ischemic heart disease (IHD). Median WHR was 1.01 for men and 0.95 for women.

After a mean follow-up period of 3.1 ± 0.81 years, 9 patients (41%) had at least one CV event. There were 9 deaths (41% mortality), and 5 out of the 9 deaths were related to CV causes (3 cardiac events, 1 stroke, and 1 ruptured abdominal aortic aneurysm). 4 out 5 CV deaths occurred in those with WHR above the gender-specific median. The other 4 deaths were due to infection or malignancy (2 with high WHR and 2 with low WHR). There were 9 first CV events; 6 occurred in those with high WHR (5 MIs and 1 stroke), and 3 occurred in those with low WHR (all MIs). 

CV risk factors and other baseline characteristics with known prognostic significance in PD patients were compared between the two groups of patients with WHR above and below the gender-specific medians. There were no significant differences in median age (61 and 62; *P* = .74), male gender (67% and 54%; *P* = .67), the presence of hypertension (89% and 77%; *P* = .62), DM (56% and 23%; *P* = .19), any smoking history (67% and 54%; *P* = .67), known IHD (44% and 15%; *P* = .18), median albumin (38 and 36 g/L; *P* = .90), peritoneal transport characteristics (high or high average 100% and 77%, *P* = .24), residual renal function (4.36 and 4.65 mL/min; *P* = .79), and transfer to HD (22 and 15%; *P* = 1.0). There were also no differences in PD modality (automated versus continuous ambulatory PD), (*P* = .41) use of icodextrin, (*P* = .36), or peritonitis rates (*P* = 1.0).

**Figure 1:**
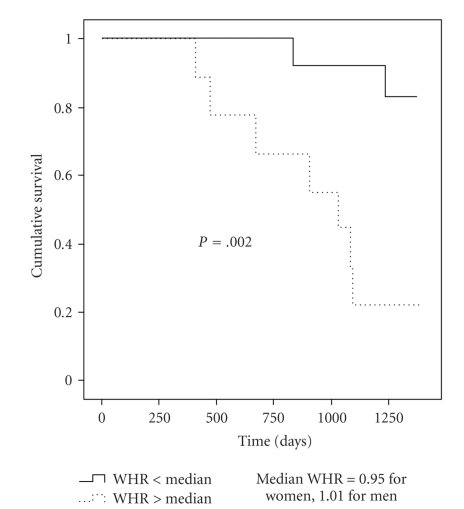
Kaplan-Meier survival curves for patients above and below the gender-specific median for WHR.

Kaplan-Meier survival analysis showed a lower cumulative survival (*P* = .002) in those with higher WHR (Figure 1). There were also increased overall CV events (*P* = .03) and CV deaths (*P* = .01) in the group with higher WHR. Kaplan-Meier survival analysis was not performed for WC and BMI due to violation of the assumption of proportionality. 

Using Cox regression, the hazard ratio (HR) for death with every 0.01 change in WHR was 1.15 (confidence interval [CI] 1.05–1.24), and for first CV event, the HR was 1.13 (CI 1.03–1.25). None of the measured baseline characteristics were significantly different between high and low WHR groups. However, because of a trend towards increased IHD and DM in the high WHR group and the known association with poor outcomes in dialysis patients, IHD and DM were entered as covariates in Cox regression models. After adjusting for IHD, HR for death was 1.17 (CI 1.05–1.30), and HR for first CV event was 1.13 (CI 1.01–1.26) in patients with high WHR. After adjusting for DM, high WHR patients had a HR for death equal to 1.16 (CI 1.05–1.28), and HR for first CV event was 1.12 (CI 1.01–1.24). 

Using logistic regression, neither WC nor BMI predicted death (odds ratio [OR} 1.06 CI 0.97–1.16 for WC and OR 1.03 CI 0.86–1.22 for BMI, resp.) or CV events (OR 1.07 CI 0.98–1.17 for WC and OR 1.06 CI 0.89–1.26 for BMI, resp.). 

WHR correlated with TNF-*α* but not with CRP or IL-6 (Table 2). WC correlated with TNF-*α* and CRP. BMI did not correlate with measured inflammatory markers. No significant correlations were found between serum inflammatory markers and DM, known IHD, cholesterol profile, or peritoneal membrane transport characteristics. 

## 5. Discussion

The main finding of this study is that increased WHR is associated with increased CV events and all-cause mortality in PD patients. To our knowledge, this is the first report of such a relationship in PD patients. These results are consistent with what has previously been identified in the general population in [4], in patients with CKD in [6], and those on HD in [7]. Our data is also consistent with results showing that abdominal obesity is a stronger adverse prognosticator than BMI and that WHR may be a better measurement of abdominal adiposity than WC [3, 6]. In the INTERHEART study, BMI showed the weakest association with myocardial infarction whereas waist circumference was intermediate between WHR and BMI [3]. In HD patients, WC was a significant predictor of CV and all-cause mortality only after adjustment for BMI whereas WHR was predictive of mortality both before and after adjustment for BMI [7]. Although there was a high mortality rate of 41% during a mean follow-up period of 3.1 years in our study, this result is in keeping with the known morality rate of the Canadian dialysis population [15, 16]. 

In the general population, higher BMI predicts mortality, but the relationship appears to differ for patients on dialysis [17]. Studies in HD patients have shown paradoxically increased survival with higher BMI [17], but results in PD patients are mixed [18]. In our study, there was no association between increased BMI and clinical outcomes. This neutral relationship was also found in several other studies in PD patients [18, 19]. Some have postulated that protective effects of elevated BMI may be neutralized by increased PD-related infection, underdialysis, or more rapid loss of residual renal function [20]. The conflicting findings may also be in part related to the inability of BMI to differentiate between fat, muscle tissue, and water. BMI reflects a combination of multiple factors including subcutaneous fat, visceral fat, muscle mass, and nutritional and hydration status [21]; these factors may have mixed and competing effects on survival [22]. Therefore, BMI does not seem to be a reliable prognostic factor in PD patients. On the other hand, WHR is primarily a measure of visceral fat which is thought to be particularly detrimental due to its proatherogenic and proinflammatory properties [10].

In this study, increased WHR is associated with higher levels of TNF-*α*, and increased WC is associated with both higher levels of TNF-*α* and CRP. These correlations are consistent with the existing hypothesis that visceral fat has important inflammatory properties. Studies in the general population have shown higher circulating levels of CRP, TNF-*α*, and IL-6 in those with increased abdominal fat when compared with BMI-matched controls [10, 23]. In one small study of PD patients, WHR was significantly associated with IL-6 but not with CRP, or TNF-*α* [12]. However, factors other than abdominal obesity such as chronic infections, volume overload, accumulation of advanced glycation end-products, and peritoneal membrane inflammation can potentially contribute to systemic inflammation in PD [24]. These factors may explain differences in results between studies and the lack of association between WHR and CRP or IL-6 in our study. 

When using gender-specific cutoffs for abdominal obesity defined by WC [25], the prevalence of abdominal obesity in this study (64%) is higher compared with that previously reported in HD patients (39%) in [7]. The mean WHR (0.98 ± 0.09) in our cohort was also higher than that reported in HD patients (0.93 ± 0.10) [7]. PD patients may be at higher risk of developing abdominal obesity compared with HD patients because of the use of glucose-based dialysis solutions. Glucose loading in PD has been postulated to contribute to hyperinsulinemia and insulin resistance [26, 27]; these factors are in turn highly associated with abdominal obesity [11, 28]. In one study, patients developed a 23% increase in intra-abdominal fat area after initiating PD, despite a lack of significant increase in weight or proportion of total fat mass [29]. Abdominal obesity may therefore be a particularly important consideration in PD patients, and this highlights the importance of clinical evaluation of glucose sparing PD prescriptions [30]. Randomized trials of glucose-sparing strategies using new dialysate solutions are underway. 

The strength of this study is the prospective design, longitudinal followup, and completeness of followup (100%). The main limitation of this study is the small sample size, which precludes extensive multivariate analysis. After adjusting for IHD and DM, WHR remained an independent risk factor for mortality and first CV event. Although there was no difference in the presence of other major CV risk factors (age, gender, diabetes, hypertension, and smoking history) or known adverse prognostic variables in PD patients (serum albumin, peritoneal transport characteristics, and residual renal function) between patients with high or low WHR, it is possible that significant differences were not detected due to the small sample size. It may be that WHR is associated with a number of other risk factors however adjustment for additional potential confounding factors in the Cox regression model was also not possible with the small sample size [14]. This study should therefore be regarded as hypothesis-generating, and the preliminary data presented here provides justification for larger studies in the future.

In conclusion, this small study suggests increased WHR may be a risk factor for CV events and mortality in PD patients. WHR is a simple measurement that can be incorporated in clinical practice, and larger studies with multivariate analysis are warranted to confirm the relationship between WHR and clinical outcomes in PD patients.

## Figures and Tables

**Table 1:** Baseline characteristics of the 22 peritoneal dialysis patients enrolled.

Variable	PD Patients *N* = 22 *n* (%) or Mean (Standard Deviation)
Age (years)	61 (SD 15.6)
Male	13 (59%)
Caucasian	21 (95%)
Time on dialysis (years)	2.1 (SD 2.8)
Diabetes Mellitus	8 (37%)
Ischemic heart disease	5 (23%)
Peripheral arterial disease	3 (14%)
Hypertension	18 (82%)
Any history of smoking	11 (50%)
Waist Circumference (cm)	101 (SD 11.5)
Men	104 (SD 12.6)
Women	97.1 (SD 8.85)
Waist-to-Hip Ratio	0.98 (SD 0.09)
Men	1.01 (SD 0.08)
Women	0.94 (SD 0.09)
BMI (kg/m^2^)	27.7 (SD 4.94)
Men	28.2 (SD 5.14)
Women	27.1 (SD 4.87)

**Table 2:** Correlations between systemic inflammatory markers and anthropomorphic measures.

	Waist-to-Hip ratio *r* (*P*)	Waist circumference *r* (*P*)	Body mass index *r* (*P*)
Inflammatory markers			
CRP	NS	0.45 (*P* = .04)	NS
TNF-*α*	0.45 (*P* = .05)	0.58 (*P* = .007)	NS
IL-6	NS	NS	NS

*r*: correlation coefficient, CRP: C-reactive protein, TNF-*α*: tumor necrosis factor-*α*, IL-6: interleukin-6, and NS: nonsignificant.
